# Advances in Genome Sequencing and Natural Rubber Biosynthesis in Rubber-Producing Plants

**DOI:** 10.3390/cimb45120585

**Published:** 2023-11-21

**Authors:** Yingchao Tan, Jie Cao, Chaorong Tang, Kaiye Liu

**Affiliations:** 1 National Key Laboratory for Biological Breeding of Tropical Crops, Hainan University, Haikou 570228, China; tanyingchao@hainanu.edu.cn (Y.T.); caojie@hainanu.edu.cn (J.C.); chaorongtang@hainanu.edu.cn (C.T.); 2 School of Breeding and Multiplication (Sanya Institute of Breeding and Multiplication), Hainan University, Sanya 572025, China; 3 Natural Rubber Cooperative Innovation Center of Hainan Province and Ministry of Education of P.R. China, Hainan University, Haikou 570228, China; 4 Yunnan Institute of Tropical Crops, Xishuangbanna 666100, China

**Keywords:** natural rubber, rubber-producing plants, genome sequencing, natural rubber biosynthesis, rubber transferase complex, rubber particles

## Abstract

Natural rubber (cis-1,4-polyisoprene, NR) is an important raw material utilized widely in the manufacturing of medical, agricultural, and industrial products. Rubber tree (*Hevea brasiliensis*) and several alternative rubber-producing plants (*Taraxacum kok-saghyz*, *Lactuca sativa*, and *Parthenium argentatum*) have the capability to produce high-quality NR. With the progress of genome sequencing, similar rubber biosynthesis pathways have been discovered among different rubber-producing plant species. NR is synthesized and stored in rubber particles, which are specialized organelles comprising a hydrophobic NR core surrounded by a lipid monolayer and membrane-bound proteins. The rubber transferase complex is considered to be the pivotal enzyme involved in catalyzing NR biosynthesis. However, the exact compositions of the RT complex in rubber-producing plants remain elusive and poorly understood. Here, we review the progress of genome sequencing, natural rubber biosynthesis, and the components of the RT complex in rubber-producing plants. We emphasize that identifying the detailed components of the RT complex holds great significance for exploring the mechanism of NR biosynthesis and accelerating molecular breeding in rubber-producing plants.

## 1. Introduction

Natural rubber (NR), which consists mainly of cis-1,4-polyisoprene, is a natural polymer compound. It stands out as the only renewable industrial material and strategic resource, often classified alongside steel, petroleum, and coal as one of the four major industrial raw materials. High-molecular-weight NR has many unique physical properties, including resilience, elasticity, resistance to abrasion and impact, efficient heat dispersion, and malleability at cold temperatures, which cannot be replaced by synthetic alternatives [1]. As a result, it finds applications in over 50,000 products, such as airplane tires, sporting goods, medical and scientific instruments, and insulated cables [2,3]. According to the report from the International Rubber Study Group (IRSG), the global demand for NR witnessed an impressive surge of 9.4% in 2021, while production increased by 5.7%. Forecasts suggest that total NR production will continue to grow, with anticipated increases of 3.5% and 3.7% in 2022 and 2023, respectively.

Latex, the cytoplasm of rubber-producing laticifers, is produced from laticifer cells or parenchymal cells in 12,500 plant species [4]. Among these, over 2500 plant species have the ability to produce *cis*-polyisoprene with a molecular weight of 10^5^ Da [2]. However, only a few plant species have been identified as viable sources of high-quality and high-molecular-weight rubber suitable for industrial-scale utilization. Rubber tree (*Hevea brasiliensis*) is a tropical tree native to South America; it belongs to the spurge family (Euphorbiaceae) and provides the sole commercial source of NR. In the year 1876, Wickham fetched 70,000 rubber tree seeds from the Amazon rainforest in Brazil and planted them in the Royal Botanic Gardens, Kew [5]. Later, rubber plantations spread to many British colonies, including Borneo, Myanmar, and India, and supplied more than 90% of the global demand for rubber [5,6]. However, at present, no plantations exist in the Amazon rainforest, and rubber can only be tapped from wild trees that grow sporadically in the rainforest. The failure to establish plantations in the Amazon basin is mainly attributed to the South American leaf blight (SALB) disease caused by the fungus *Pseudocercospora ulei* [7]. Currently, the rubber tree is mainly planted in Southeast Asia, specifically Thailand and Indonesia. The restrictions of climatic factors, long growth cycles, narrow genetic bases, and susceptibility to fungal infections make the global supply of natural rubber from *H. brasiliensis* insecure [8,9,10,11,12]. Therefore, many countries around the world have recognized the significance of seeking alternative sources of NR. Several of the most promising alternative rubber-producing plants, including *Taraxacum kok-saghyz* Rodin (TKS), *Lactuca sativa* L. (Lettuce), and *Parthenium argentatum* (Guayule), were identified after assessing a large number of plant species [8]. Notably, the properties of NR in these plant species are similar to those of *H. brasiliensis* [13]. TKS belongs to the family Asteraceae, subfamily Cichorioideae, and is extensively distributed in temperate zones [14]. It accumulates NR in the laticifer cells of its roots, with a dry rubber content of up to 20% [15]. Moreover, TKS exhibits strong environmental adaptability, a short life cycle, and a mature transgenic system, positioning it as a model plant for rubber production and research. However, self-incompatibility and a high degree of heterozygosity pose obstacles to its domestication [8,15]. Lettuce, belonging to the Asteraceae family, is a temperate annual or biennial herb with abundant latex. It is an easily cultivated, self-pollinating annual plant with a 4–5 month life cycle, which makes it an ideal candidate for research in rubber production [16]. The relatively low NR content in latex is the primary limiting factor for the development and utilization of lettuce as a rubber-producing plant. Guayule is a perennial shrub that grows 0.3–0.9 m tall, inhabits arid and semi-arid areas, and can survive in desert environments with temperatures exceeding 40 °C and severe aridity [1,17]. NR derived from guayule bark parenchymal cells mainly consists of cis-1,4-polyisoprene with a molecular weight of 1280 kD, comparable to that of *H. brasiliensis*. Additionally, guayule rubber lacks allergenic proteins, making it suitable for specialized use in the medical field [18]. In a 2-year-old guayule plant, NR is present in the bark, stem, root, and leaf, with an average content of 8% of the dry weight [19]. However, guayule NR is consistently contaminated with resin, which necessitates its removal through solvent extraction to enhance the properties of the NR [8].

Over the past decade, significant progress has been made in the study of rubber-producing plants. Here, we reviewed the progress in the field of genome sequencing, the biosynthesis pathways of natural rubber, the components and regulated mechanisms of the rubber transferase complex, as well as future research directions for rubber-producing plants.

## 2. Progress in the Genome Sequencing of Rubber-Producing Plants

Progress in researching and understanding the mechanism of NR biosynthesis has been slow, failing to keep pace with the rapidly growing global demand for industrial NR consumption. One important reason for this is the lack of high-quality genomic information. The genome sequencing progress of rubber-producing plants is summarized in Table 1. In 2013, the draft genome of the rubber tree (RRIM600) was first sequenced using a whole-genome shotgun approach, with an assembled 2.15 Gb genome [20]. A total of 68,955 predicted genes were identified, including the key genes associated with rubber biosynthesis, rubberwood formation, disease resistance, and allergenicity. In 2016, two rubber tree genomes, RRIM600 and Reyan7-33-97, were reported, with their genome sizes being significantly reduced to 1.55 Gb and 1.47 Gb, respectively [21,22]. This reduction was achieved through the integration of Illumina sequencing data with either PacBio or BAC clone sequencing data. Both studies discovered that the capacity of *H. brasiliensis* to produce high levels of latex can be attributed to the expansion of rubber biosynthesis-related genes as well as the high expression of these genes in latex. In 2017, the genome of the BPM24 clone was assembled using Illumina short reads and PacBio long-read data [23]. Notably, a long-range “Chicago” assembly technique was employed to scaffold the preliminary assembly, resulting in 1.26 Gb of assembled sequences. Moreover, 363 Mb sequences of the genome were mapped to 18 linkage groups by using an SNP-based genetic map. In 2020, the first chromosome-level genome of the rubber tree cultivar GT1 was assembled using single-molecule real-time sequencing (SMRT) and chromosome conformation capture (Hi-C) technologies [24]. The GT1 genome has a size of 1.47 Gb, and it is estimated to contain 44,187 protein-coding genes. Additionally, this study has identified numerous candidate domestication genes involved in rubber biosynthesis between cultivated and wild rubber trees. In 2023, a high-quality, chromosome-level genome sequence of the wild rubber tree (MT/VB/25A 57/8) was reported by combining Illumina sequencing data, SMRT sequencing data, Bio-Nano data, and Hi-C data [25]. A total of 35,318 predicted protein-coding genes were identified from the 1.72 Gb genome sequences. Furthermore, the population genomic analysis found 361 selection signatures that align with 245 genes and 155 significant markers associated with latex yield that correspond to 326 candidate genes. During the same year, a high-quality genome of the elite rubber tree cultivar CATAS8-79 was assembled by integrating PacBio CLR sequencing data, Hi-C data, and Bio-Nano data [26]. The CATAS8-79 genome has a size of 1.58 Gb, with 1.55 Gb assigned to 18 chromosomes, indicating a remarkably high level of sequence continuity. A total of 38,595 high-confidence gene models were predicted and functionally annotated in the CATAS8-79 genome. The continuous improvement in genomic integrity has not only facilitated the genome-assisted selection breeding of rubber trees but also expedited the domestication of other rubber-producing plants.

The first draft genome of TKS (TK1151) was reported in 2017, with an assembled 1.29 Gb genome predicted to contain 46,731 protein-coding genes, including 102 candidate genes involved in the NR biosynthesis pathway [15]. Following that, the genome was significantly improved by integrating PacBio SMRT sequencing, Bio-Nano optical mapping, and Hi-C technologies, thereby generating a more continuous genome of 1.07 Gb in 2021 [27]. Furthermore, through the comparison of the *T. mongolicum* (TM5) genome, a species closely related to TKS, gene family expansion events were identified in the NR biosynthesis pathway in TK1151. The genome of lettuce was assembled in 2017 using the Illumina whole-genome shotgun method [16]. The assembly comprises 2.38 Gb and encompasses 38,919 gene models, covering approximately 88% of the estimated genome size. In 2020, a tetraploid guayule was sequenced using Illumina NovaSeq platform, resulting in an assembled genome size of 2.93 Gb and consisting of 1,070,894 scaffolds. The genomic data, expressed sequencing tags, and plastid genome data of the tetraploid guayule have been retrieved in the Guayule Genomic Resources (https://probes.pw.usda.gov/Guayule/index.html). In 2021, Bridgestone and NRGene jointly announced the successful sequencing and assembly of a diploid guayule genome. However, the detailed genome sequence has not yet been released, and only limited sequence information can be obtained through sequence alignment [28]. Progress in the genome sequencing of alternative rubber-producing plants provides the basis for elucidating the molecular mechanism of natural rubber biosynthesis.

## 3. Natural Rubber Biosynthesis

### 3.1. Biosynthesis of the Building Block IPP and Initiator Molecules

**Figure 1:**
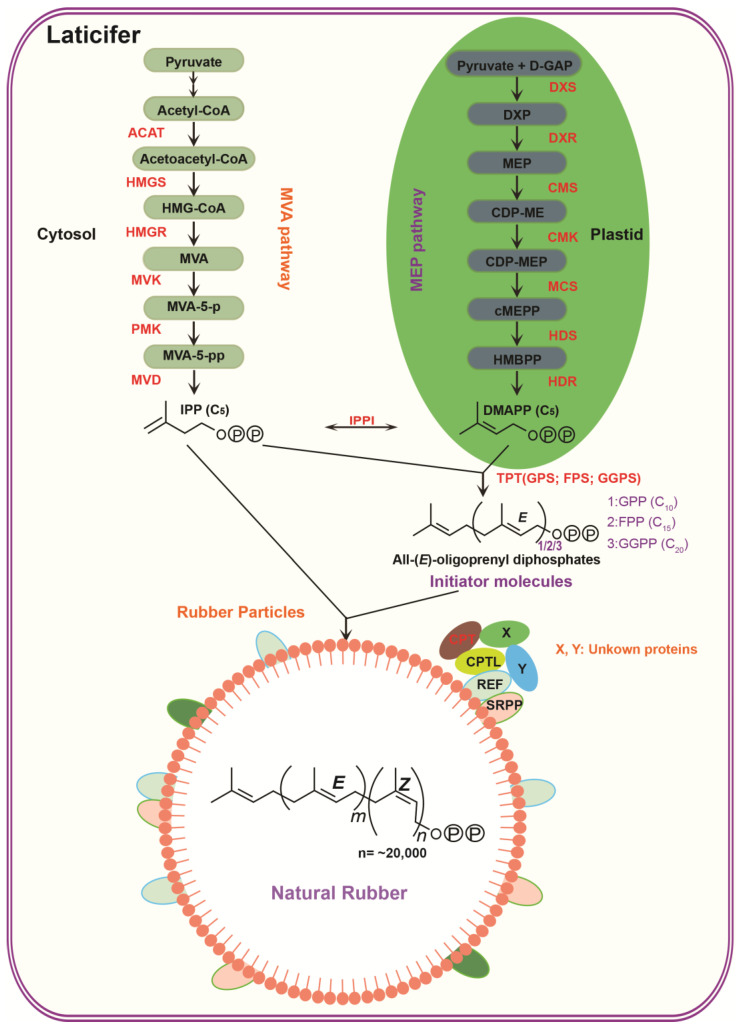
Natural rubber biosynthesis. ACAT: acetyl-CoA acetyltransferase; HMGS: 3-hydroxy-3-methyl-glutaryl-coenzyme synthase; HMGR: HMG-CoA reductase; MVK: mevalonate kinase; PMK: phosphomevalonate kinase; MVD: mevalonate diphosphate decarboxylase; DXS: 1-deoxy-D-xylulose 5-phosphate synthase; DXP: 3-phosphate into 1-deoxy-D-xylulose 5-phosphate; DXR: 1-deoxy-D-xylulose 5-phosphate reductorisomerase; CMS: 2-C-methyl-D-erythritol 4-phosphate cytidyltransferase; CMK: 4-diphosphocytidyl-2-C-methyl-D-erythritol kinase; MCS: 2-C-methyl-Derythritol 2,4-cyclodiphosphate synthase; HDS: 1-hydroxy-2-methyl-2-(E)-butenyl 4-diphosphate synthase; HDR: 1-hydroxy-2-methyl-2-(E)-butenyl 4-diphosphate reductase; IPPI: isopentenyl diphosphate isomerase; GPS: geranyl pyrophosphate; FPS: farnesyl diphosphate synthase; GGPS: geranylgeranyl pyrophosphate synthase; IPP: isopentenyl pyrophosphate; CPT: cis-prenyltransferase.

IPP is a precursor for the biosynthesis of NR and various isoprenoid compounds. Rubber-producing plants utilize two distinct pathways to synthesize the key rubber monomer, isopentenyl pyrophosphate (IPP): the mevalonate (MVA) pathway in the cytosol and the 2-C-methyl-D-erythritol-4-phosphate (MEP) pathway in plastids (Figure 1) [6,29]. Acetyl-CoA is enzymatically converted into IPP through a series of reactions, which involves six major steps catalyzed by specific enzymes in the MVA pathway [30,31,32,33,34]. These enzymes include acetyl-CoA acetyltransferase (ACAT), 3-hydroxy-3-methyl-glutaryl-coenzyme synthase (HMGS), HMG-CoA reductase (HMGR), mevalonate kinase (MVK), phosphomevalonate kinase (PMK), and mevalonate diphosphate decarboxylase (MVD). Among them, HMGS and HMGR have been identified as key rate-limiting enzymes. The MEP pathway employs seven consecutive enzymes to convert pyruvate and D-glyceraldehyde-3-phosphate into IPP [35]. The initial step of the MEP pathway, catalyzed by 1-deoxy-D-xylulose 5-phosphate synthase (DXS), is rate-limiting. This step converts the precursors pyruvate and glyceraldehyde 3-phosphate into 1-deoxy-D-xylulose 5-phosphate (DXP). Afterward, the formation of the final IPP is catalyzed by six enzyme relays, including 1-deoxy-D-xylulose 5-phosphate reductoisomerase (DXR), 2-C-methyl-D-erythritol 4-phosphate cytidyltransferase (CMS), 4-diphosphocytidyl-2-C-methyl-D-erythritol kinase (CMK), 2-C-methyl-Derythritol 2,4-cyclodiphosphate synthase (MCS), 1-hydroxy-2-methyl-2-(E)-butenyl 4-diphosphate synthase (HDS), and 1-hydroxy-2-methyl-2-(E)-butenyl 4-diphosphate reductase (HDR). Studies have shown that metabolites can flow between the cytoplasmic MVA pathway and the plastid MEP pathway [21,36,37]. Therefore, it is speculated that the MEP pathway can serve as an alternative pathway for supplying IPP during natural rubber biosynthesis (Figure 1).

Subsequently, isopentenyl diphosphate isomerase (IPPI) catalyzes the conversion of a portion of IPP into DMAPP. The synthesis of initiator molecules such as geranyl pyrophosphate (GPP), farnesyl pyrophosphate (FPP), and geranylgeranyl pyrophosphate (GGPP) is generated through the consecutive condensation of IPP and DMAPP [8]. This condensation is catalyzed by a group of prenyl pyrophosphate synthase enzymes, including geranyl diphosphate synthase (GPS), farnesyl diphosphate synthase (FPS), and geranylgeranyl pyrophosphate synthase (GGPS). Finally, by sequentially condensing 15,000–30,000 IPPs on the initial molecules, a substantial chain of natural rubber with a high molecular weight is generated (Figure 1).

### 3.2. Rubber-Synthesizing Organelle: –Rubber Particle (RP)

Natural rubber biosynthesis primarily takes place on the surface of rubber particles. Therefore, analyzing the structure, compositions, and surface proteins of rubber particles constitute the primary focus of NR biosynthesis research [6,8,38,39,40]. RPs are located within the cytoplasm of laticifer cells, which can be found in the phloem of the rubber tree and TKS, and the parenchyma cells of the guayule [29]. Fresh latex can be divided into three distinct layers through ultracentrifugation: the upper layer consisting of RPs, the middle layer containing cytoplasmic serum, and the bottom layer comprising lutoids [41]. Based on the composition of rubber particles, the ontogenesis of RPs in laticifer cells may originate from either the endoplasmic reticulum or the Golgi apparatus. The hydrophobic core of an RP is primarily composed of cis-polyisoprene, which constitutes more than 90% of the RP’s weight [6]. Surrounding this core is an outer layer comprised of a lipid monolayer, along with proteins and other components. The protein compositions of rubber particles can be divided into three types: (1) proteins that embed transmembrane domains in the lipid monolayer; (2) proteins without transmembrane domains that are covalently bound to lipid molecules; and (3) proteins that form complexes with the first two types. Typically, these proteins form complexes or act synergistically during natural rubber biosynthesis [41,42]. RPs exhibit a globular structure, with their diameter varying among different species: 0.02–2.0 μm in *H. brasiliensis*, 0.2–10 μm in *T. kok-saghyz*, 0.5–2.0 μm in *P. argentatum*, and 1.6–6.5 μm in Ficus species [1,43,44,45,46,47]. According to the diameter distribution, the RPs from *H. brasiliensis* can be classified into small rubber particles (SRPs) and large rubber particles (LRPs), with 94% being SRPs and 6% being LRPs in fresh latex [22,43,45,48]. Notably, it is precisely this 6% of LRPs by number that comprise 93% of the rubber by volume in latex. Interestingly, in vitro assays have shown that SRPs exhibit significantly higher rubber biosynthesis activity compared to LRPs [46,49,50].

### 3.3. The Core Enzyme for Rubber Synthesis: Rubber Transferase Complex

The rubber transferase complex, which can either be bound to the surface or embedded within the single layer of rubber particles, is the most vital enzyme complex responsible for the catalytic synthesis of natural rubber. Therefore, identifying the components of the rubber transferase complex is the basis for studying the mechanism of rubber biosynthesis. *cis*-prenyltransferase (CPT) is responsible for catalyzing the condensation of an allylic prenyl diphosphate to IPP and plays a crucial role in the biosynthetic activity of NR (Figure 1). Furthermore, CPT has the ability to terminate IPP condensation by effectively detecting the carbon chain length of the resulting product. This termination process depends on the structures of the catalytic site and the hydrophobic space, which facilitate the accommodation of the hydrophobic prenyl products [6]. Two CPT genes, namely *Hevea brasiliensis rubber transferase 1* (*HRT1*) and *HRT2*, were isolated from the latex of *H. brasiliensis* [51,52]. When expressed as recombinant proteins in the *E. coli* system, HRT1 and HRT2 did not exhibit any enzymatic activity [51]. However, the addition of buffer-washed rubber particles significantly enhanced their activity. The observation suggests that HRTs play a pivotal role as key enzymes in the biosynthesis of NR, working in coordination with other unknown essential cofactors. Nogo-B receptor (NgBR) proteins serve as partners of *cis*-prenyltransferases, commonly referred to as *cis*-prenyltransferase-like (CPTL) proteins. Homologs of NgBR have been isolated from NR-producing plants, including *H. brasiliensis* (*HbCPTL*), *L. sativa* (*LsCPTL*), and *T. brevicorniculatum* (*TbRTA*) [42,53,54]. Furthermore, NgBR interacts with the HRTs and the rubber elongation factor (REF) in the rubber tree. Therefore, it is also referred to as the HRT-REF bridging protein (HRBP) [42,55]. REFs are highly abundant expressed proteins that are closely bound to rubber particles. In *H. brasiliensis*, the transcript levels of *REFs* show a positive correlation with latex yields [56]. The REF interacts with the small rubber particle protein (SRPP) and itself, indicating its capability to form homodimer and heterodimer structures for functional purposes [57]. A cell-free translation-coupled system assay on washed rubber particles (WRPs) indicates that the REF protein may be involved in the stabilization or maintenance of RPs, thereby reducing the coagulation of latex [6]. Homologs of the SRPP have been identified in both rubber-producing plants and non-rubber-producing plants, and they are designated as the SRPP family or stress-related protein family. The classification of REF proteins as members of the SRPP family is based on their possession of a conserved REF domain [58]. The SRPPs are located on the surface of rubber particles due to their interactions with the REF, while their binding to the rubber particles is not as tight as that of the REF. Additionally, the identification of three proteins as constituents of a membrane-bound complex was accomplished by employing labeled ^3^H-Bz-GPP(*p*). However, the specific protein sequence remained unidentified [59]. The identification of the components comprising the rubber transferase complex will facilitate further exploration into the in vitro synthesis of natural rubber and provide valuable insights for the synthesis of natural rubber using bioreactors.

## 4. Research Progress in NR Biosynthesis Regulation

The genome assembly of *H. brasiliensis* revealed 94 genes related to rubber biosynthesis which belong to 20 gene families [22]. Among these genes, 18 are associated with the mevalonate (MVA) pathway, 22 with the methylerythritol phosphate (MEP) pathway, 15 with cytosolic initiator synthesis, and 39 with putative rubber particle-associated rubber elongation genes. Similarly, the genome assembly of *T. kok-saghyz* revealed a total of 102 candidate genes related to rubber biosynthesis [15]. Among these, 40 genes are involved in all six steps of the MVA pathway, 23 genes are involved in all seven steps of the MEP pathway, 19 genes are associated with initiator synthesis, and 20 genes are associated with rubber-particle-associated rubber elongation proteins. The key proteins associated with rubber particles include rubber elongation factors (REFs), *cis*-prenyltransferases (CPTs), *cis*-prenyltransferase-like (CPTL) proteins, and small rubber particle proteins (SRPPs) [42,51,57,60,61]. Hence, these proteins have been widely identified and studied in rubber-producing plants, shedding light on the regulatory mechanisms of rubber biosynthesis.

*cis*-prenyltransferases (CPTs), belonging to the prenyltransferase family, have been shown to possess the activity of incorporating IPP into polyprenyl chains, playing a key role in NR biosynthetic activity. HRT1 and HRT2 are two *Hevea* rubber transferases predominantly expressed in latex. They possess all five highly conserved regions crucial for the catalytic function and substrate binding of *cis*-prenyl chain elongating enzymes [51,52]. As mentioned earlier, HRT proteins purified from the *E. coli* system exhibited no CPT activity. However, the heterologous expression of *HRT1* and *HRT2* in *Arabidopsis* cell cultures resulted in long-chain CPT activity but failed to form NR [62]. Moreover, homologues of CPT have been isolated from other NR-producing plants such as *T. koksaghyz*, *L. sativa*, and *E*. *characias*, but none of them have been enzymatically identified as having rubber transferase activity [47,53,63]. These findings suggest that the CPT proteins require additional factors to exhibit their activity. The silencing of *CPTs* in *T. brevicorniculatum* through RNA interference (RNAi) resulted in a decrease in NR content, suggesting their involvement in NR biosynthesis [64]. Knocking out *LsCPT3* via CRISPR/Cas9 in lettuce significantly reduced the rubber content in latex [13]. Notably, the growth and development of the mutants are unaffected, which indicates that NR does not have a significant physiological function in lettuce. Interestingly, both the native *LsCPT3* and heterologous *CPTs* from guayule (*PaCPT3*) and goldenrod (*ScCPT3*) proved the ability of complementing the mutants, resulting in the production of NR with a molecular weight (MW) exceeding 1 million Da in the *lscpt3* background. In contrast, native goldenrod plants can only produce NR with a MW of 0.09 million Da.

CPTLs are homologous to the human Nogo-B receptor (NgBR) and play a crucial role in rubber biosynthesis. They are primarily localized in the endoplasmic reticulum (ER) of non-rubber-producing eukaryotes and can also be found in both the ER and rubber particles of rubber-producing plants [53,60]. Although lacking the conserved catalytic residues commonly found in CPT proteins, the C-terminal RxG motif of CPTL has been demonstrated to be essential for pentenyl-transferase activity [65]. The rubber transferase activator, TbRTA, in *T. brevicorniculatum* interacts with TbCPTs located on the surface of rubber particles, thereby contributing to the formation of the rubber transferase complex [66]. Knocking out the *TbRTA* resulted in a significant impairment of rubber synthesis, providing further evidence for the indispensability of *TbTRA* (*TbCPTL*) within the rubber transferase complex. HbCPTL/HbHRBP interacts with HbHRT1/2 and HbREF, potentially serving as a bridging component between HRTs and the REF, facilitating the formation of the rubber transferase complex [29,42,60]. HRT1 exhibits rubber transferase activity on WRP, whereas the HRBP or REF did not demonstrate such activity. The co-expression of the HRBP or the HRBP and REF with HRT1 significantly enhances rubber transferase activity but has no impact on the length of the product chain [42]. This evidence indicates that the REF may play a role in stabilizing the HRT1-HRBP complex. In addition, the ternary protein complex consisting of HRT1, the HRBP, and the REF has also been observed in *Nicotiana benthamiana*, where it is localized on the ER and ER-derived particles.

In addition to CPTs, rubber elongation factors (REFs) and small rubber particle proteins (SRPPs) are also crucial for the biosynthesis of high-molecular-weight natural rubber [51]. The REF and SRPP are homologous proteins within the stress-related protein family, typically exhibiting high transcriptional abundance in the latex of rubber-producing plants. REF subfamily members exhibit variability in their N-terminal region and possess a relatively short C-terminal region beyond the REF domain. Conversely, SRPP subfamily members feature a short N-terminal region and display variability in their C-terminal region [58]. Knocking down *REF* transcripts in *T. brevicorniculatum* results in a significant reduction in rubber content, which correlates with lower levels of TbCPT proteins in the latex. However, the molecular mass and stability of the rubber particles remain unaffected [67]. The overexpression of *TkSRPP3* resulted in an increase in the dry rubber content of TKS, whereas RNAi *TkSRPP3* led to a significant reduction in both rubber content and rubber molecular weight [68]. In *T. brevicorniculatum*, the RNAi lines of *TbSRPPs* exhibit a 40–50% reduction in dry rubber content, with no significant impact on molecular weight and polydispersity [69]. In contrast, knockdown lines of *LsSRPP4* and *LsSRPP8* show no effect on rubber content, molecular weight, and polydispersity in lettuce [54].

## 5. Conclusions and Perspectives

Although rubber trees face various challenges, such as long breeding cycles, narrow genetic diversity, limited planting areas, rising labor costs of harvesting, and the threat of South American leaf blight, they remain presently almost the sole commercial source of natural rubber [10,11,12,70,71]. In recent years, there has been a significant acceleration in the genome sequencing of rubber trees, resulting in the release of high-quality genome assemblies from a number of cultivars and wild germplasm [23,24,25,26]. As a result, genome sequencing research in rubber trees has reached a cutting-edge level. Meanwhile, genome research has advanced the studies of population genetics in rubber trees, such as parental mapping populations and genome-wide association study (GWAS) populations, and led to notable research breakthroughs. The complete genome sequences also provide a solid foundation for identifying various genes associated with rubber biosynthesis. Subsequently, a series of core catalytic enzymes and regulatory genes related to rubber biosynthesis were identified in latex and thereby enhanced our understanding of the rubber biosynthesis process. However, the genetic transformation of the rubber tree remains a significant challenge, rendering it a less-than-ideal candidate for investigating the underlying regulatory mechanisms of rubber production.

Several alternative rubber-producing plants, including *T. koksaghyz*, *L. sativa*, and *P. argentatum*, are being developed to address the problems faced by rubber trees and to expand sources of natural rubber. Genetic transformation systems have been established in these plants, with each having its own advantages in natural rubber production. Interestingly, these plants share nearly identical rubber-producing pathways as rubber trees and retain the homologs of reported rubber transferase complex components, such as *CPT*, *CPTL*, *REF*, and *SRPP*. The overexpression and knock-out of these genes in alternative rubber-producing plants have demonstrated their critical roles in rubber transferase activity and rubber biosynthesis. However, in vitro rubber biosynthesis experiments have shown that the combination of all these proteins lacks rubber transferase activity, suggesting that unknown essential components of the rubber transferase complex remain to be identified. Therefore, the identification of new components of the rubber transferase complex is crucial to gaining deeper insight into the process of rubber biosynthesis. In addition, rubber transferase complex members tend to exhibit specific high expression levels. Furthermore, the transcription factors and other regulatory components responsible for the predominant expression of the rubber transferase complex in rubber-producing laticifer cells or parenchyma cells have been poorly understood. We believe that identifying new components of the rubber transferase complex and their regulatory factors will contribute to both the understating of rubber production in alternative rubber-producing plants and the molecular breeding of high-yielding rubber clones.

## Figures and Tables

**Table 1:** Summary of genome sequencing progress in rubber-producing plants.

Species	Accessions	Genome Size	Number of Protein-Coding Genes	N50 Scaffold Length	Sequencing Libraries/Platforms	Year	Reference
*Hevea brasiliensis*	RRIM600	2.15 Gb	68,955	2.97 Kb	Roche/454, Illumina, and SOLiD	2013	[20]
	RRIM600	1.55 Gb	84,440	67.24 Kb	Illumina and mate-pair sequencing	2016	[21]
	Reyan7-33-97	1.47 Gb	43,792	1.28 Mb	Illumina GA2 and Hiseq 2000, and mate-pair sequencing	2016	[22]
	BPM24	1.26 Gb	43,868	96.80 Kb	Roche 454 GS FLX, Illumina HiSeq 2000, and PacBio RSII	2017	[23]
	GT1	1.47 Gb	44,187	152.7 Kb	Illumina HiSeq 2000, PacBio SMRT, Illumina HiSeq 2500, and Hi-C	2020	[24]
	Wild rubber tree (MT/VB/25A 57/8)	1.72 Gb	35,318	102.00 Mb	SMRT, Oxford Nanopore, Illumina HiSeq X Ten, and Hi-C	2023	[25]
	CATAS8-79	1.58 Gb	38,595	11.21 Mb	BGISEQ-500, PacBio CLR, Hi-C, BioNano	2023	[26]
*Taraxacum kok-saghyz*	TK1151	1.29 Gb	46,731	100.21 Kb	PacBio RSII, and Illumina HiSeq 2500	2017	[15]
		1.07 Gb	45,224	131.57 Mb	PacBio SMRT, BioNano optical-mapping, and Hi-C	2021	[27]
*Taraxacum mongolicum*	TM5	781.19 Mb	45,553	96.94 Mb	PacBio SMRT, BioNano optical-mapping, Hi-C, and Illumina whole-genome shotgun	2021	[27]
*Lactuca*	*Lactuca sativa*	2.38 Gb	38,919	1.80 Mb	Illumina, Chicago, HiRise	2017	[16]
*Parthenium argentatum*	Diploid guayule	1.52 Gb	~40,000	22 Kb	Illumina, Illumina MiSeq, Roche 454, and Roche 454 GSFLX +	2018	[28]
	Tetraploid guayule	2.93 Gb	-	-	-	2020	-

## Data Availability

Not applicable.
